# Robust visual SLAM algorithm based on target detection and clustering in dynamic scenarios

**DOI:** 10.3389/fnbot.2024.1431897

**Published:** 2024-07-23

**Authors:** Fubao Gan, Shanyong Xu, Linya Jiang, Yuwen Liu, Quanzeng Liu, Shihao Lan

**Affiliations:** ^1^School of Electrical and Information Engineering, Anhui University of Science and Technology, Huainan, China; ^2^School of Artificial Intelligence, Anhui University of Science and Technology, Huainan, China

**Keywords:** visual SLAM, target detection, clustering, dynamic scenarios, feature points

## Abstract

We propose a visual Simultaneous Localization and Mapping (SLAM) algorithm that integrates target detection and clustering techniques in dynamic scenarios to address the vulnerability of traditional SLAM algorithms to moving targets. The proposed algorithm integrates the target detection module into the front end of the SLAM and identifies dynamic objects within the visual range by improving the YOLOv5. Feature points associated with the dynamic objects are disregarded, and only those that correspond to static targets are utilized for frame-to-frame matching. This approach effectively addresses the camera pose estimation in dynamic environments, enhances system positioning accuracy, and optimizes the visual SLAM performance. Experiments on the TUM public dataset and comparison with the traditional ORB-SLAM3 algorithm and DS-SLAM algorithm validate that the proposed visual SLAM algorithm demonstrates an average improvement of 85.70 and 30.92% in positioning accuracy in highly dynamic scenarios. In comparison to the DynaSLAM system using MASK-RCNN, our system exhibits superior real-time performance while maintaining a comparable ATE index. These results highlight that our pro-posed SLAM algorithm effectively reduces pose estimation errors, enhances positioning accuracy, and showcases enhanced robustness compared to conventional visual SLAM algorithms.

## 1 Introduction

Simultaneous Localization and Mapping (SLAM) is a technique where a robot utilizes its own sensors to perceive surrounding information, enabling self-localization without prior environmental knowledge (Khnissi et al., 2022; Sahili et al., 2023). SLAM can be categorized into two main types: laser SLAM and visual SLAM. Visual SLAM employs cameras as data acquisition sensors, providing richer image information. Therefore, it has emerged as a prominent research and application field (Li et al., 2021; Xing et al., 2022; Xu et al., 2023).

The visual SLAM approaches can also be broadly categorized into two main types: feature point method and direct method. In the mainstream feature point method, images captured by the camera are extracted and matched to locate corresponding features on a given map (Eslamian and Ahmadzadeh, 2022; Jiang et al., 2023). Building upon the foundation of traditional ORBSLAM2 (Mur-Artal and Tardós, 2017), Campos et al. (2021) proposed the ORBSLAM3 as an exemplar of the feature point method. The DTAM system proposed by Newcombe et al. (2011) serves as the precursor of the direct method, aligning the entire image for obtaining a dense map and camera pose, thereby enabling real-time dense 3D reconstruction. The LSD-SLAM system proposed by Engel et al. (2014) leverages grayscale information from images to achieve localization and construct a semi-dense point cloud map. The DSO system proposed by Matsuki et al. (2018) is a visual odometer that combines both the direct and sparse methods, utilizing an optimization algorithm to minimize the photometric errors to realize construction of a sparse point cloud map.

In addition to input data, alternative approaches have been put up to address the SLAM issues. These techniques can be broadly classified into two categories: filtering-based and optimization-based techniques (Huang et al., 2013; Liang et al., 2014; Tu et al., 2022). Particle filters and Extended Kalman filters are two instances of the filtering-based technique. Such methods are employed due to the prevalence of noise and inconsistencies in sensor data, allowing for modeling of diverse sources of noise and their impacts on measurements. The second set, the optimization-based technique, has gained significant popularity due to their superior stability, efficiency, robustness, and scalability compared to the filtering-based technique. In these approaches, measurements are typically represented as a graph structure wherein nodes correspond to the robot’s poses and edges denote spatial constraints between different poses (Chen et al., 2022).

The fundamental assumption underlying most early SLAM systems was that the camera constituted the sole moving object within the field of view (Soares and Meggiolaro, 2018; Shen et al., 2021), while the surrounding environment remained predominantly static (Li and Lee, 2016; de Backer et al., 2023). However, both in-door and outdoor scenarios present non-ideal conditions for these frequently encountered situations. Efforts have been made to treat such moving objects, particularly people, as outliers and exclude them from environmental representations. To enhance positioning accuracy in dynamic context, several other SLAM systems employ frame-works that integrate the SLAM system with target tracking and detection (Newcombe et al., 2015; Kim and Kim, 2016; Li and Lee, 2017; Ai et al., 2020; Cui and Ma, 2020; Cheng et al., 2021; Wu et al., 2021).

In recent years, significant achievements have been made in the field of computer vision through the utilization of deep learning algorithms. Currently, target detection algorithms based on deep learning can be mainly categorized into two types: the traditional two-step approaches including R-CNN (Hmidani and Ismaili Alaoui, 2022) and Fast R-CNN (Girshick, 2015), and the more modern end-to-end methods such as YOLO (Han et al., 2023) and SSD (Xiong and Fan, 2021). While the former exhibits high precision, they suffer from limited real-time performance; whereas the latter have greatly improved in real-time capabilities but struggle with detecting small tar-gets accurately. However, with continuous development in deep neural networks, there has been a notable enhancement in image-based target detection accuracy (Hary and Mandala, 2022).

Therefore, those deep learning approaches have been introduced into visual SLAM in recent years. For example, to mitigate the impact of dynamic objects on SLAM, Yu et al. (2018) incorporated semantic segmentation and optical flow techniques into ORB-SLAM2, proposing a DS-SLAM algorithm to alleviate the influence of moving individuals in complex environments. Additionally, they introduced the construction of a semantic octree map to enhance positioning and mapping accuracy. However, it should be noted that semantic segmentation entails significant computational time, hindering real-time performance and potentially leading to feature tracking failures due to insufficient feature points post-dynamic removal. Bescos et al. (2018) pro-posed the DynaSLAM algorithm, which incorporates dynamic target detection and background restoration functions into traditional ORB-SLAM. Additionally, Mask R-CNN (He et al., 2017) is utilized for instance segmentation of dynamic objects. This algorithm improves the accuracy of pose estimation localization; however, it also increases corresponding computational time, rendering it unsuitable for real-time SLAM applications. Zhong et al. (2018) developed the Detect-SLAM system by integrating the SSD (Single Shot Multi Box Detector) target detection network with a SLAM system to identify objects in image sequences using a pre-trained target detection network. Dynamic feature points are removed during the ORB feature extraction stage to significantly enhance the accuracy and robustness of SLAM in dynamic environments. Ran et al. (2021) introduced RS-SLAM as a robust semantic RGB-D SLAM system that improves region of interested extraction accuracy by incorporating context information based on Bayesian update to modify segmentation results. Furthermore, it is capable of constructing a clean static semantic OctoMap in a dynamic environment. Some experts and scholars have done some research in the field of YOLO and SLAM, such as, Tian et al. (2022) proposed a SLAM system based on ORB-SLAM2 for dynamic environment, Based on RGB-D camera, the system uses YOLOX-S to detect dynamic objects and combines depth information to filter dynamic points. Tang et al. (2023) introduced a visual SLAM framework designed for dynamic indoor environments. Cong et al. (2024) proposed a dynamic visual SLAM (SEG-SLAM) system based on the orientated FAST and rotated BRIEF (ORB)-SLAM3 framework and you only look once (YOLO)v5 deep-learning method. You et al. (2022) proposed a novel multimodal semantic SLAM system (MISD-SLAM), which removes the dynamic objects in the environments and reconstructs the static background with semantic information.

To enhance the positioning accuracy and real-time performance of visual SLAM, this study proposes a robust visual SLAM algorithm that integrates target detection and clustering in dynamic scenarios by incorporating the lightweight YOLOv5 net-work. The primary research contributions are as follows:

1. The original backbone network in YOLOv5 is substituted with a Ghost light-weight module to effectively reduce network parameters, while simultaneously incorporating the CBAM attention mechanism into the Backbone to enhance its ability in capturing important information. Additionally, the K-means clustering algorithm is employed to determine anchor frame sizes that align with the detection scale within the detection network. By introducing detection layers and expanding detection scales, notable improvements are achieved in terms of the network’s detection performance;
2. The target detection module has been integrated into the front end of SLAM, utilizing an improved YOLOv5 algorithm for enhanced performance.

## 2 Visual SLAM algorithm integrating target detection and clustering in dynamic scenarios

### 2.1 Visual SLAM mathematical model

SLAM requires the robot to perceive environmental information through its in-stalled sensors and subsequently accomplish self-localization map construction. This paper utilizes data from the camera sensors, whereby when the robot detects a land-mark point $y_{j}$ at a specific location $x_{k}$, it generates an observation datum $z_{k,j}$ that can be represented by an abstract function h:


(1) $$z_{k,j}=h\left(y_{j},x_{k},v_{k,j}\right)$$

Equation 1 is the observation equation, where $v_{k,j}$ represents the observation noise.

It can also be described by an abstract function f:


(2) $$x_{k}=f\left(x_{k-1},u_{k},w_{k}\right)$$

Equation 2 is the motion equation, where $x_{k-1}$ and $x_{k}$ denote the states of the robot at k−1 and k respectively; $u_{k}$ denotes the input of the motion sensor; and $w_{k}$ denotes the noise added in the process.

The SLAM process can be summarized into Equation 3:


(3) $$\{\begin{array}{l} x_{k}=f\left(x_{k-1},u_{k},w_{k}\right)\\ z_{k,j}=h\left(y_{j},x_{k},v_{k,j}\right) \end{array}$$

where u represents the measured motion value and z the sensor reading. The above two equations explain the methodology for solving the positioning problem (estimation x) and the mapping problem (estimation y) when u and z are known. This is formulated as a state estimation problem, typically addressed through extended Kalman filter and nonlinear optimization techniques. Considering the superior performance of nonlinear optimization in visual SLAM, this paper adopts a nonlinear optimization scheme.

In this approach, all variables to be estimated are consolidated into a single state variable, the process is shown in Equation 4:


(4) $$x=x\left(x_{1},x_{2}\cdots ,x_{N},y_{1},y_{2},\cdots ,y_{N}\right)$$

When the input data u and the observation data z are known, the conditional probability distribution of state x is P(x|,u|,z).

### 2.2 Traditional visual SLAM framework

**Figure 1:**
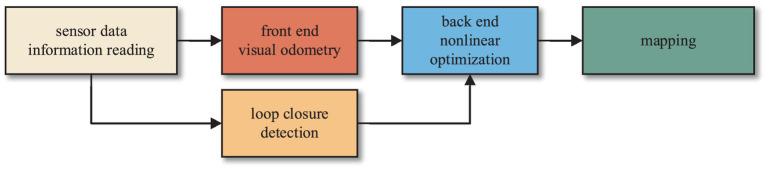
Classic visual SLAM framework.

The classic SLAM system is usually composed of a front end and a back end. The primary function of the front end is to construct the map by utilizing the sensor data to obtain initial state estimates and establish constraints between states. On the other hand, the back end employs the nonlinear optimization to mitigate cumulative errors. The algorithm framework is illustrated in Figure 1.

Firstly, feature points are extracted from the images captured by the camera. Subsequently, feature matching is performed on the two consecutive images to eliminate mismatched feature points. Then, estimation of pose changes for the mobile robot at the current moment is conducted based on these adjacent images. The pose changes estimated by the visual odometry typically represent short-term variations and are prone to cumulative errors as the robot moves. Therefore, closed-loop detection and back-end optimization are employed in the SLAM system to reduce these cumulative errors.

**Figure 2:**
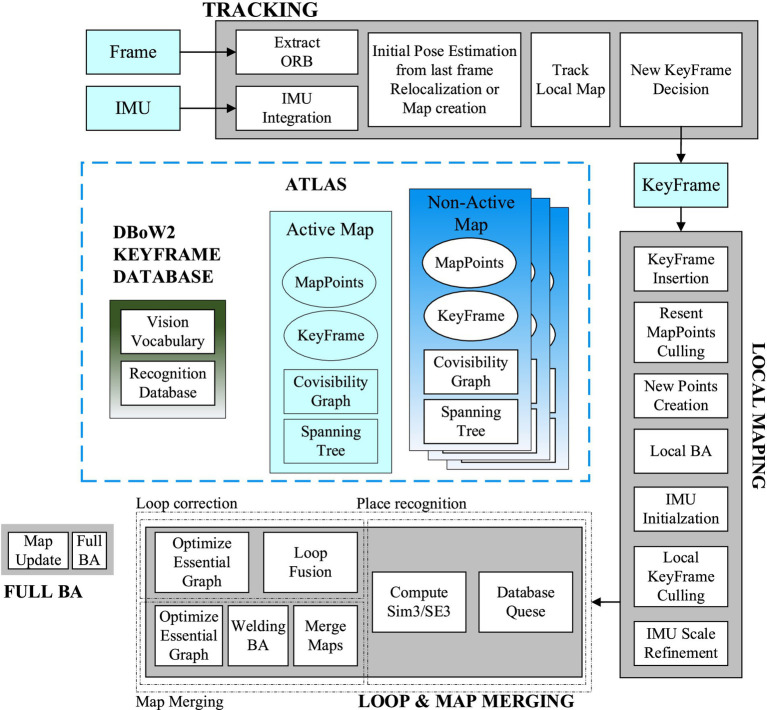
Threads and structure diagram of ORB-SLAM3 system.

ORB-SLAM3 is the pioneering feature-based tightly coupled VIO system. The Threads and structure diagram of ORB-SLAM3 is shown in Figure 2. It incorporates three concurrent threads for tracking, local mapping, closed-loop detection, and map fusion. The tracking thread extracts and matches ORB feature points while estimating the relative pose between two frames by minimizing the reprojection error. The local mapping thread integrates new keyframes and Map Points into the active map, eliminates redundancy, and updates the map using BA within a sliding window. The closed-loop detection and map fusion thread identifies keyframes to detect potential closed loops, and continuously corrects the accumulated drift errors through pose graph optimization. Finally, the global BA considers medium- to long-term matches from a closed-loop detection to provide an optimized MAP estimate.

### 2.3 Lightweight target detection algorithm based on YOLOv5s

**Figure 3:**
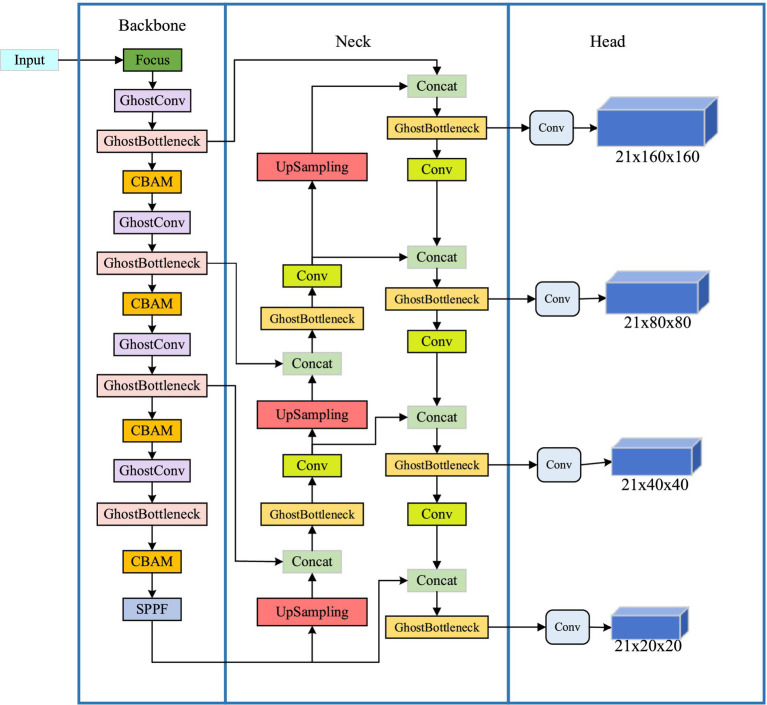
Improved YOLOv5s network structure diagram.

The YOLO series serves as an exemplary representative of the first-order target detection algorithms. Compared with traditional algorithms, the YOLO algorithm exhibits a simple structure, faster detection speed, and higher detection accuracy in detecting targets. Building upon YOLOv4, YOLOv5 further enhances its network architecture, training strategy, and data augmentation techniques to achieve improved speed and accuracy. Due to its lightweight characteristics and low memory usage, YOLOv5 proves advantageous for application scenarios involving mobile devices or resource-constrained systems. Among the YOLOv5 series models, YOLOv5s stands out with faster runtime performance while imposing lower hardware requirements; thus making it more suitable for deployment on mobile terminals. Considering that the indoor dynamic environment primarily consists of large targets and mobile devices have limited computing power, this section proposes an enhanced lightweight target detection algorithm based on YOLOv5s network to cater to the demands of real-time target detection in dynamic scenarios. The improvements include: (1) Replacing common convolutions with more lightweight Ghost convolutions to reduce computational complexity and enhance system performance; (2) Incorporating CBAM attention mechanism into Backbone for improved information capture capability; (3) Introducing the K-means clustering algorithm for better effects, adding detection layers and scales to enhance network’s detection performance. The modified structure of the YOLOv5s network is illustrated in Figure 3.

#### 2.3.1 Replace ghost convolution

The original backbone network is partitioned, and then the feature maps of three scales of 128×80×80, 256×40×40, and 512×20×20 are obtained through three rounds of downsampling. To reduce computational load, a lightweight module Ghost Bottleneck is introduced to replace the BottleneckCSP convolution module in the backbone network. The Ghost module primarily ensures network detection accuracy while employing less computationally intensive linear operations instead of the original convolution operation, achieving feature map generation via a convolution kernel. Furthermore, the depth wise convolution is employed to perform linear operations on each channel of the feature map for channel expansion, thereby effectively con-ducting hierarchical convolution processing on the input feature map.

**Figure 4:**
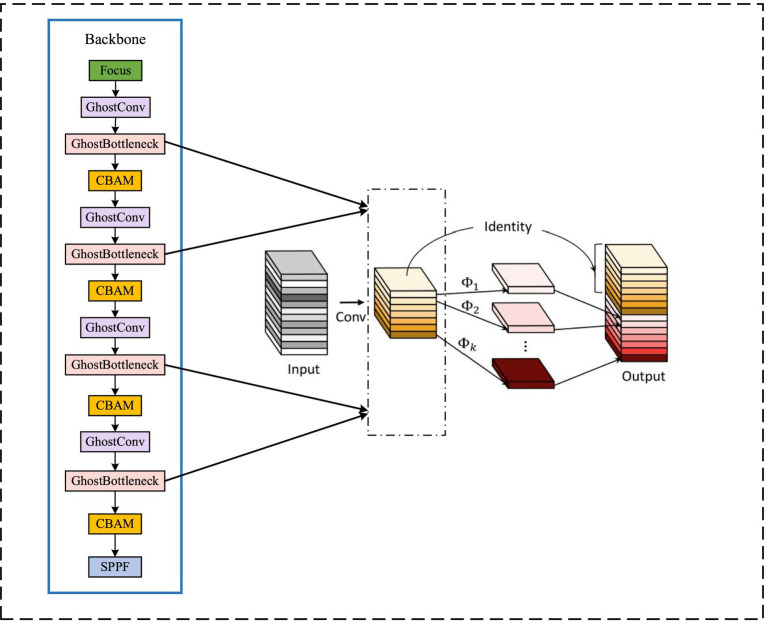
Ghost backbone network structure diagram.

The Ghost lightweight module structure diagram is depicted on the left in Figure 4, while the improved backbone network structure is presented on the right. By incorporating a lightweight network, computational load of the network is effectively reduced.

The ordinary convolution operation, the parameter $NUM_{parameters}$, and the calculation FLOPs expression are respectively:


(5) $$NUM_{parameters}=k_{size}\times k_{size}\times c_{in}\times c_{out}$$

(6) $$FLOPs=c_{out}\times h_{out}\times w_{out}\times c_{in}\times k_{size}\times k_{size}$$

Where: $k_{size}$ is the size of the convolution kernel; $c_{in}$ and $c_{out}$ are the number of input channels and output channels, respectively; $h_{out}$ and $w_{out}$ are the height and width of the output feature map, respectively.

The expressions of parameter $G\_NUM_{parameters}$ and computation G_FLOPs of the GhostMoudle network are:


(7) $$G\_NUM_{parameters}=1\times 1\times c_{in}\times \frac{c_{out}}{2}\times k_{size}\times k_{size}\times \frac{c_{out}}{2}$$

(8) $$\begin{array}{l} G\_FLOPs=\frac{c_{out}}{2}\times h_{out}\times w_{out}\times c_{in}\times 1\times 1+\\ \;\;\;\;\;\;\;\;\;\;\;\;\;\;\;\;\;\;\;\;\;\;\;\;\;\;\;\;\;\;\;\;\frac{c_{out}}{2}\times h_{out}\times w_{out}\times k_{size}\times k_{size} \end{array}$$

Through the comparison Eqs. (5–8), it can be seen that the calculation amount and parameter number of the GhostBottleneck network are 1/(2$k_{size}$×$k_{size}$) + 1/(2$c_{in}$) times that of the ordinary convolution operation, which can achieve the purpose of network lightweight.

The network proposed in this paper fully leverages the feature map generated during the sampling process on the backbone network. Building upon the original YOLOv5 three-layer detection layer, we combine the downsampling-generated feature map with its corresponding scale feature map from the head to form a minimum scale detection layer.

#### 2.3.2 Add K-means clustering

The YOLOv5-based detection network in this study enhances the detection net-work by integrating the detection characteristics of target objects. In the original detection network, the anchor frame size is predetermined, and target objects are detected across different feature maps. Specifically, a smaller anchor frame is employed to detect small target objects on larger feature maps that contain more intricate details, while the larger anchor frame is set to detect large target objects on smaller feature map.

The detection network is designed to detect human objects, and the size of different types of objects varies greatly, requiring calculation of new anchor frame sizes ac-cording to the annotation information in the dataset. To obtain a more suitable anchor frame size for detection scale matching, the K-means clustering algorithm is utilized to divide annotated anchor frames in the dataset into clusters that match network detection scales.

The specific process of the K-means algorithm is as follows. Firstly, a value is randomly selected from the sample as the clustering center $C_{1}$, and the minimum IoU distance d(x) between all samples and the existing clustering center is calculated. The clustering center $C_{1}$ is selected according to the probability by using the Eq. (9). Repeat this step until K cluster centers are found. For each sample $x_{i}$ in the data set, the IoU distance from $x_{i}$ to K cluster centers is calculated, and it is divided into the category corresponding to the cluster center with the smallest distance. According to the division result, K clustering centers are recalculated by using Eq. (10), and the operation is repeated until the position of the clustering center does not change, and the final clustering center is output. Through the K-means clustering algorithm, the anchor box suitable for the TUM RGB-D dataset is finally generated.


(9) $$C_{i}=\left(1/\left|C_{1}\right|\right)\underset{x\in C_{1}}{\sum }x$$

(10) $$p=d{\left(x\right)}^{2}/\underset{x\in B}{\sum }d{\left(x\right)}^{2}$$

#### 2.3.3 Add CBAM attention mechanism

The Convolutional Block Attention Module (CBAM) attention mechanism ad-dresses both the channel and spatial dimensions in the feature graphs obtained through convolutional operations. In the channel attention module, the spatial dimension of the feature maps is compressed, assigning weights to different channels based on their respective features. Similarly, within the spatial attention module, the feature map is compressed along the channel dimension while obtaining an attention map with varying weights for different spatial positions.

**Figure 5:**
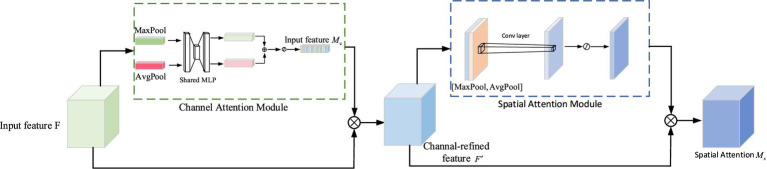
CBAM module structure diagram.

The processing of the input feature map is illustrated in Figure 5, where CBAM exhibits a structured architecture comprising two interconnected components: the channel attention module and the spatial attention module. The input feature map is divided into two branches. One branch represents the feature map from the previous stage, which undergoes channel attention module to acquire attention weights of equal dimensions. The other branch corresponds to the original feature map, and their respective outputs are multiplied together to yield the input for the next stage. In the next stage, it further bifurcates into two branches: one for generating a feature map with spatial attention, and the other for a feature map with channel attention for the preceding stage. By multiplying these branch-specific feature maps, a composite feature map encompassing both channel and spatial attention is ultimately obtained.

The following will introduce the access attention structure and the spatial attention structure, respectively.

Where: F is the input feature map; $M_{c}$ is the feature map output after passing through the channel attention structure; $F^{\prime }$ represents the result of the multiplication of $M_{c}$ and F; $M_{s}$ represents the feature map output after the CBAM attention mechanism; AvgPool represents the average pooling operation; MaxPool represents the maximum pooling operation; MLP represents multi-layer perceptron (fully connected layer); σ is the Sigmoid activation function.

As shown in the figure, the channel attention module in the CBAM attention mechanism extracts the spatial information of the feature map by summing the input feature map F through global average pooling and global maximum pooling to obtain two-channel feature maps $F_{\max}^{c}$ and $F_{avg}^{c}$. Then, the shared network hidden layer MLP will process the two features passed in turn. Once the function σ is activated, the attention channel feature map $M_{c}$ will be obtained. The two-layer parameters in the multi-layer perception model are represented by $W_{0}$ and $W_{1}$. The Channel Attention Module-specific calculation formula is shown in Equation 11:


(11) $$\begin{array}{l} M_{s}\left(F\right)=\sigma \left(MLP\left(AvgPool\left(F\right)\right)+MLP\left(MaxPool\left(F\right)\right)\right)\\ \;\;\;\;\;\;\;\;\;\;\;\;\;\;\;\;\;\;\;=\sigma \left(W_{1}\left(W_{0}\left(F_{avg}^{c}\right)\right)+W_{1}\left(W_{0}\left(F_{\max}^{c}\right)\right)\right) \end{array}$$

The Spatial Attention Module module in the CBAM attention mechanism first performs global maximum addition and global mean addition on each channel to obtain two H×W×1 feature maps and then performs channel splicing. After 7×7 convolution, the dimension is reduced to H × W × 1. Next, the spatial attention feature is generated by the Sigmoid activation function, and finally, the final feature output is obtained by multiplying the input feature map. The specific calculation formula of the Spatial Attention Module module is shown in Equation 12:


(12) $$\begin{array}{l} M_{s}\left(F\right)=\sigma \left(f^{7*7}\left(\left[MaxPool\left(F\right);AvgPool\left(F\right)\right]\right)\right)\\ \;\;\;\;\;\;\;\;\;\;\;\;\;\;\;\;\;\;=\sigma \left(f^{7*7}\left(\left[F_{avg}^{s};F_{\max}^{s}\right]\right)\right) \end{array}$$

where σ represents the sigmoid function; $F_{\max}^{c}$ and $F_{avg}^{c}$ represent the maximum pooling operation and the average pooling operation in the Channel Attention Module, respectively. $F_{\max}^{s}$ and $F_{avg}^{s}$ represent the maximum pooling operation and the average pooling operation in the Spatial Attention Module, respectively. $f^{7*7}$ denotes a convolution kernel of size 7 × 7.

#### 2.3.4 Add detection scale

During the target detection process, the network is required to simultaneously detect targets of different sizes, and the proportions of these targets can significantly impact the detection accuracy of the network model. In our dataset, mice constitute a relatively small proportion compared to other objects with larger proportions. As we increase the depth of the network, both the detailed and semantic information about target objects undergo continuous changes. Shallow layers in the network tend to pro-vide better semantic information for small target objects; however, deeper layers may diminish their semantic information while causing loss of details. On the other hand, larger target objects necessitate a deeper network to capture their semantic information effectively. To better improve the detection accuracy of convolutional neural networks, we design a network structure tailored o the size characteristics specific to four types of target objects present in the TUM RGB-D dataset: person, chair, computer, and keyboard.

As shown in Figure 3, the network architecture consists of three components arranged from left to right: the network backbone for image information acquisition, the network neck for feature fusion, and the network head for computational processing to detect target object category and position. Moreover, a four-scale detection hierarchy is incorporated into the design of the network to enable compatible detection of both large and small target objects. By effectively fusing the shallow detail information with the deep semantic information, valuable image details extracted from feature maps are retained, thereby facilitating simultaneous detection of target objects with significant proportion differences and ultimately enhancing overall network detection performance.

### 2.4 Robust visual SLAM based on target detection and clustering

**Figure 6:**
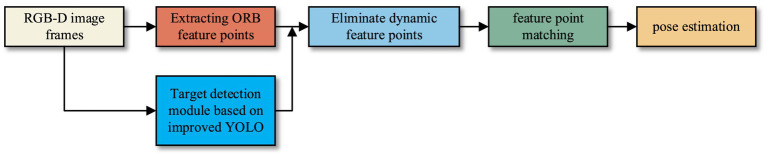
The improved SLAM tracking thread in this paper.

To enhance the positioning accuracy and robustness of the traditional ORB-SLAM system in dynamic environments, we integrate the above improved YOLOv5 algorithm as an object detection module into SLAM. The target detection module is incorporated into the tracking thread to identify and eliminate the dynamic ORB feature points on moving targets, ensuring that only the static feature points contribute to the pose estimation. The improved SLAM tracking thread in this paper is shown in Figure 6.

## 3 Results and discussion

### 3.1 Experiment setting

We implement all experiments on a computer with Intel Core i7-11700 CPU, Nvidia GTX 3060 GPU, 32 GB RAM. The operating system is Ubuntu 18.04 with ROS Melodic.

The input image size is 640×480, the initial learning rate is 0.01, the learning rate momentum is 20.8, the weight attenuation coefficient is 0.0005, the batch_size is 4, the workers is 1, and the epoch is 300.

### 3.2 Experimental datasets

The TUM dataset, widely employed for evaluating SLAM performance in dynamic scenarios, is selected for this experiment. The TUM RGB-D dataset comprises two types of scenes: high dynamic scenes and low dynamic scenes. The high dynamic scene is denoted as “walking,” while the low dynamic scene is referred to as “sitting.” Each type of dynamic scene is further divided into four image sequences: halfsphere, xyz, rpy, and static. These sequences represent distinct camera movements during image acquisition: traversing in a 1 m hemisphere, moving along coordinate axes, rotating on rolling, pitch and yaw axes, or remaining stationary. In the sitting (S) sequence, two individuals are seated at a table engaged in conversation and gesturing, resulting in minimal movement. In the walking (W) sequence, two individuals simultaneously traverse the background and foreground before eventually taking their seats in front of the table. This dataset exhibits highly dynamism, posing significant challenges for conventional SLAM systems.

### 3.3 Lightweight target detection algorithm verification

#### 3.3.1 Dataset

Considering that the primary dynamic objects in the indoor scene are predominantly individuals, this experiment aims to assess the efficacy of the enhanced Yolov5s algorithm by selecting a total of 1,000 images from the TUM dataset, specifically from the ‘people’ category, for training and testing purposes. All tests are conducted 5 times, and the final results are averaged.

#### 3.3.2 Performance evaluation

The performance of the target detection algorithm is usually evaluated by mean average precision (mAP), which reflects the accuracy performance of the model under different recall rates. A higher mAP value indicates that the model can maintain high accuracy even under a high recall rate. Therefore, the higher the mAP value, the better the performance of the model. The calculation procedure is outlined in Equation 13:


(13) $$mAP=\frac{1}{n}\overset{n}{\underset{i=1}{\sum }}AP_{i}$$

Where m represents ‘mean’, and AP represents the average accuracy of a certain type of sample. In this paper, ablation experiments are carried out to compare the impacts of different strategies (replacing Ghost convolution, incorporating CBAM attention mechanism, and adding a detection layer) on model performance. The evaluation metric used is mAP@0.5 with an IoU threshold set at 0.5.

The results in Table 1 demonstrate that the replacement of the original convolution with Ghost convolution in the Yolov5s algorithm leads to a reduction in model parameters to 4.88 M, an increase in detection speed by 7 FPS, and a slight decrease in detection accuracy by 2.4%. Furthermore, incorporating CBAM attention mechanism and detection layer can further enhance the detection accuracy. Compared with the original algorithm, our proposed algorithm increases the mAP by 7%, reduces model size by 28.9%, and enhances detection speed by 6FPS. These improvements effectively address the demand for lightweight target detection algorithms on mobile devices as they not only improve the detection accuracy but also meet the real-time requirements.

**Table 1:** Comparative results of ablation experiments.

Models	mAP%	Parameters/MB	FPS
YOLOv5s	85.5	7.06	22
Ghost	83.1	4.88	29
CBAM	86.7	7.23	19
Detection layer	91.8	7.42	20
Improved YOLOv5s	92.5	5.02	28

#### 3.3.3 Dynamic target detection experiment

**Figure 7:**
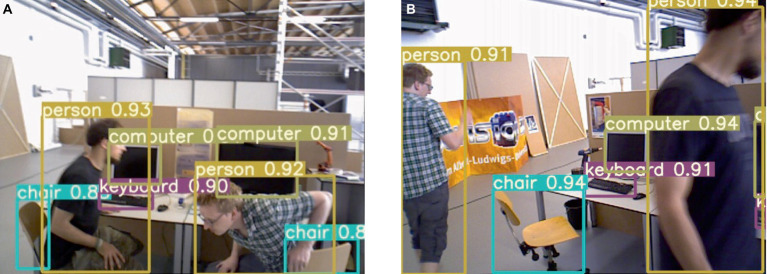
Example of improved YOLOv5s detection effect.

**Figure 8:**
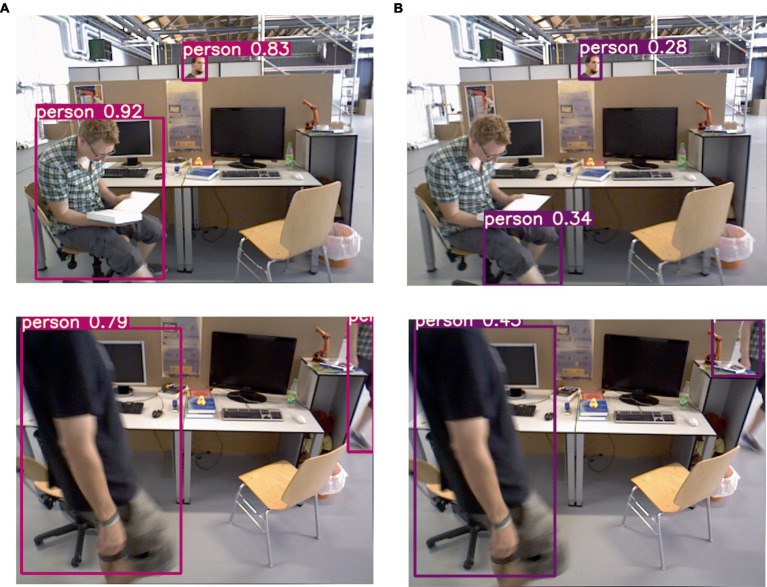
Results of detecting people in motion.

The improved lightweight YOLOv5s network is employed for target detection, and the resulting detection performance is illustrated in Figure 7. As depicted, the category of the detection result is presented within the figure. In dynamic scenes, humans are considered as high-mobility targets; thus, the target detection network is configured exclusively for human recognition. The comparison test results before and after adding K-means clustering are shown in Figure 8, (a) listed as the test results after adding K-means, and (b) listed as the test results without adding K-means. It can be observed that on the one hand, the large target can be completely detected, and on the other hand, it still has high confidence when only the exposed head, arm or leg parts of the human body are visible.

**Figure 9:**
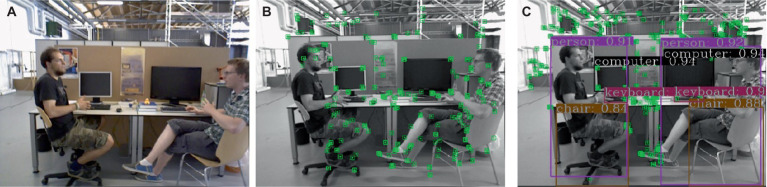
The comparison of feature point extraction between the proposed algorithm and the ORB-SLAM3 algorithm.

The comparative performance results between our algorithm and the traditional ORB-SLAM3 algorithm on the f3 dataset are illustrated in the Figure 9: (a) is the original image, (b) is the feature points extracted by the original ORB-SLAM3, and (c) is the feature points extracted by our proposed algorithm.

### 3.4 Pose estimation accuracy experiment

#### 3.4.1 Comparison of trajectory error results

Absolute Trajectory Error (ATE) is commonly employed to evaluate the positioning accuracy of the SLAM systems. In our pose estimation error analysis experiment, we utilize the evo tool to evaluate and compare the camera pose CameraTrajectory.txt estimated by the ORB-SLAM system with the true pose groundtruth.txt given by the dataset. The ATE enables us to quantify the disparity between the true and estimated values of the camera pose, thereby assessing global consistency of the trajectory. We compute the root mean square error (RMSE), median (Median), mean (Mean), and standard deviation (Std) as evaluation metrics for quantifying errors.

**Figure 10:**
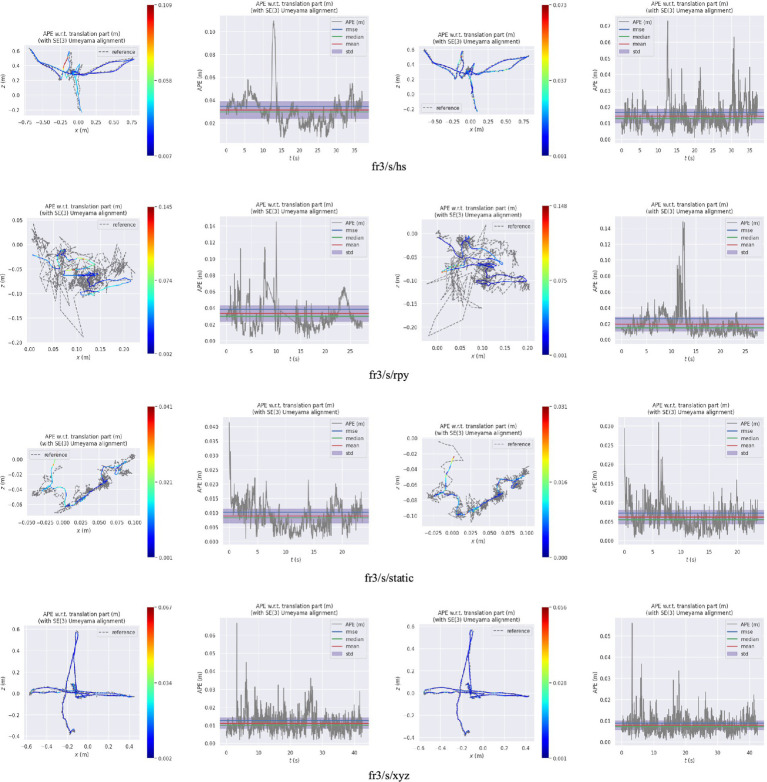
The trajectory and error map in a low dynamic scene (the first and second columns are, respectively, the trajectory and error of ORB-SLAM3; the third and fourth columns are, respectively, the trajectory and error of the proposed algorithm).

**Figure 11:**
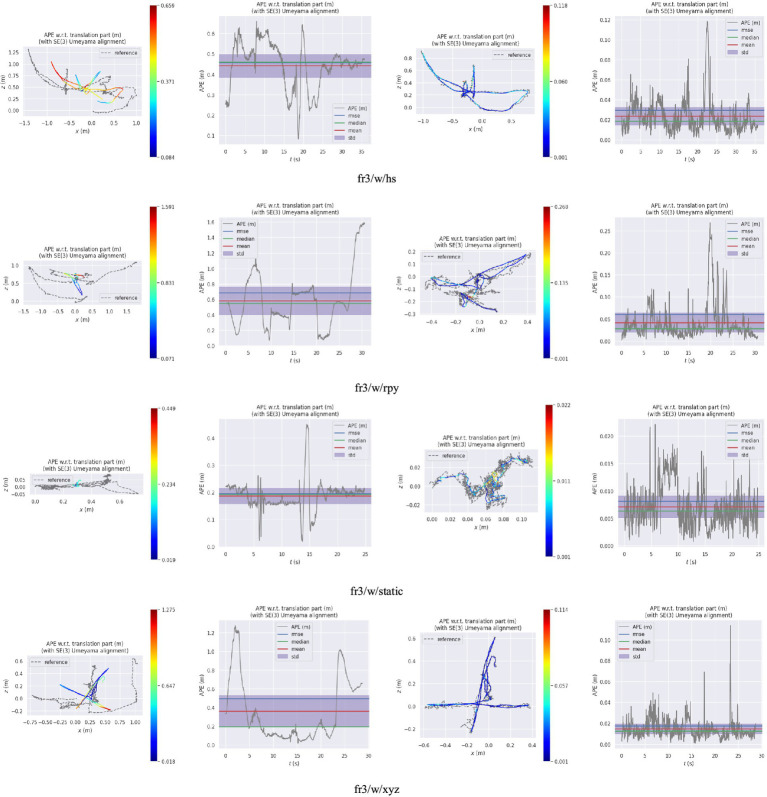
The trajectory and error map in a high dynamic scene (the first and second columns are, respectively, the trajectory and error of ORB-SLAM3; the third and fourth columns are, respectively, the trajectory and error of the proposed algorithm).

Figure 10 presents the comparison results between the real trajectory and the trajectory estimated by, respectively, the ORB-SLAM3 and our algorithm in a low dynamic scene. Figure 11 demonstrates such comparison results in a high dynamic scene. The first and second columns of these figures represent the trajectory and error of ORB-SLAM3 respectively, while the third and fourth columns are the trajectory and error of our proposed algorithm. The gray dashed line represents the true camera trajectory, whereas the colored line represents the estimated trajectory. In the high dynamic scene, it can be observed from the trajectory map that our proposed algorithm exhibits minimal deviation from the actual gray trajectory with higher stability. Furthermore, compared to ORB-SLAM3 algorithm, our proposed approach demonstrates significantly improved positioning accuracy and robustness.

The results presented in Tables 2, 3 demonstrate that when compared with the ORB-SLAM3 system, the average improvement rates of RMSE, Median, and Mean for the four high dynamic test datasets are up to 85.70, 95.58 and 95.45%, respectively. Upon analyzing the images processed by the target detection network and observing the system’s operational state, it is found that in the walking_static dataset, a significant number of image frames have figures occupying an excessively large frame area. Consequently, this leads to a reduced detection of static feature points. Under the four low dynamic test datasets, the average improvement rates of RMSE, Median, and Mean are 35.16, 41.73, and 38.71% respectively, less significant than those in high dynamic scenes. In the low dynamic sequences, the majority of objects exhibit relatively fixed positions and attitudes, posing challenges in identifying objects or regions with significant dynamic characteristics within the sequence. Consequently, the availability of feature points suitable for tracking is severely limited.

**Table 2:** Comparison of absolute trajectory error between ORB-SLAM3 and our proposed algorithm.

Sequence	ORB-SLAM3				Ours			
	RMSE	Median	Mean	Std	RMSE	Median	Mean	Std
fr3/w/hs	0.4566	0.4607	0.4422	0.1136	0.0296	0.0189	0.0236	0.0178
fr3/w/rpy	0.6833	0.5470	0.5831	0.3563	0.0364	0.0218	0.0292	0.0218
fr3/w/static	0.1947	0.1926	0.1862	0.0568	0.0815	0.0063	0.0071	0.0040
fr3/w/xyz	0.4952	0.1940	0.3604	0.3397	0.0174	0.0123	0.0146	0.0095
fr3/s/hs	0.0347	0.0298	0.0313	0.0149	0.0166	0.0129	0.0143	0.0084
fr3/s/rpy	0.0385	0.0296	0.0333	0.0194	0.0264	0.0148	0.0194	0.0178
fr3/s/static	0.0102	0.0082	0.0089	0.0049	0.0072	0.0055	0.0062	0.0036
fr3/s/xyz	0.0123	0.0099	0.0109	0.0056	0.0089	0.0072	0.0078	0.0043

**Table 3:** The absolute trajectory error performance improvement of the proposed algorithm compared to ORB-SLAM3.

Sequence	RMSE	Median	Mean	Std
fr3/w/hs	93.5173	95.8976	94.6631	84.3301
fr3/w/rpy	94.6729	96.0146	94.9923	93.8816
fr3/w/static	58.1407	96.7290	96.1869	92.9578
fr3/w/xyz	96.4863	93.6598	95.9490	97.2034
fr3/s/hs	52.1614	56.7114	54.3131	43.6242
fr3/s/rpy	31.4286	50.0000	41.7417	8.2474
fr3/s/static	29.4118	32.9268	30.3371	26.5306
fr3/s/xyz	27.6423	27.2727	28.4404	23.2143

The visual SLAM algorithm proposed in this paper is compared with other SLAM algorithms in dynamic scenes, such as DynaSLAM and DS-SLAM. The experimental results and comparisons are presented in Table 4, where the bold fonts indicate the best performance while the underlined texts represent the second-best results. The top-performing results in the tables are primarily attributed to DynaSLAM and the algorithm proposed in this paper. Compared with the DS-SLAM algorithm, our pro-posed algorithm exhibits significantly improved localization accuracy and runtime efficiency in high dynamic scenarios, resulting in a reduction of 91.8 and 29.6%, respectively, for RMSE of absolute trajectory error on walking_xyz and walking_static sequences. Compared with the DynaSLAM algorithm, our algorithm achieves similar positioning accuracy while demonstrating faster runtime and processing speed due to avoiding the time-consuming image processing involved in the Mask R-CNN instance segmentation utilized by DynaSLAM. Table 5 lists the time required for the four algorithms to process each frame of picture under the walking_xyz sequences. So, our algorithm achieves a good balance between accuracy and real-time performance, and it can effectively deal with the effects of moving objects on the stability of SLAM systems in a dynamic environment.

**Table 4:** Comparison of absolute trajectory errors of different algorithms.

Sequence	ORB-SLAM3	DynaSLAM	DS-SLAM	YOLO + SLAM	Ours
fr3/w/hs	0.4566	**0.0226**	0.0303	0.0335	0.0296
fr3/w/rpy	0.6833	0.0400	0.4442	0.0423	**0.0364**
fr3/w/static	0.1947	0.0090	**0.0081**	0.0136	**0.0081**
fr3/w/xyz	0.4952	**0.0135**	0.0247	0.0196	0.0174
fr3/s/hs	0.0347	0.0179	-	0.0176	**0.0166**
fr3/s/rpy	0.0385	0.0482	-	0.0332	**0.0264**
fr3/s/static	0.0102	**0.0058**	0.0065	0.0078	0.0072
fr3/s/xyz	0.0123	0.0134	-	0.0098	**0.0089**

**Table 5:** Comparison of tracking time of different algorithms.

Algorithm	Time (ms)
ORB-SLAM3	16.9
DynaSLAM	1,020
DS-SLAM	300
Ours	21.2

## 4 Conclusion

This paper presents a robust visual SLAM algorithm that combines target detection and clustering in dynamic scenarios to address the challenge of reduced positioning accuracy and robustness by dynamic objects. Our algorithm employs a lightweight target detection algorithm based on YOLOv5s for real-time detection of dynamic objects. We replace the backbone network of YOLOv5s with a Ghost lightweight module to reduce network parameters, while adding CBAM attention mechanism to enhance the network’s ability to capture important information. Additionally, we incorporate the K-means clustering algorithm to obtain anchor frame size matching the detection scale in the detection network, thereby improving network detection performance through addition of detection layers and increased detection scales.

The proposed algorithm is evaluated on the TUM RGB-D dataset in this study. The test results demonstrate a significant reduction of 85.7 and 30.9%, respectively, in the absolute trajectory error of our algorithm as compared with the ORB-SLAM3 and DS-SLAM algorithms under high dynamic scenarios. Moreover, the proposed algorithm exhibits comparable positioning accuracy to DynaSLAM but with improved computational efficiency. These findings validate the superior positioning accuracy and robustness of our proposed visual SLAM algorithm that integrates target detection and clustering techniques for dynamic scenes, thereby highlighting its promising practical applications. Future research directions may involve exploring more advanced semantic segmentation networks and multi-sensor fusion approaches to further optimize and enhance the algorithm’s performance.

## Data availability statement

The original contributions presented in the study are included in the article/supplementary material, further inquiries can be directed to the corresponding author.

## Author contributions

FG: Methodology, Software, Writing – original draft, Writing – review & editing. SX: Conceptualization, Writing – review & editing. LJ: Software, Writing – review & editing. YL: Investigation, Writing – review & editing. QL: Validation, Writing – review & editing. SL: Supervision, Writing – review & editing.
